# Evolving evidence in adult idiopathic intracranial hypertension: pathophysiology and management

**DOI:** 10.1136/jnnp-2015-311302

**Published:** 2016-02-17

**Authors:** Susan P Mollan, Fizzah Ali, Ghaniah Hassan-Smith, Hannah Botfield, Deborah I Friedman, Alexandra J Sinclair

**Affiliations:** 1 Birmingham Neuro-Ophthalmology Unit, Ophthalmology Department, University Hospitals Birmingham NHS Trust, Queen Elizabeth Hospital Birmingham, Birmingham, UK; 2 Neurometabolism, Institute of Metabolism and Systems Research, College of Medical and Dental Sciences, University of Birmingham, Birmingham, UK; 3 Neurology Department, University Hospitals Birmingham NHS Trust, Queen Elizabeth Hospital Birmingham, Birmingham, UK; 4 Department of Neurology and Neurotherapeutics, University of Texas Southwestern Medical Center, Dallas, Texas, USA; 5 Department of Ophthalmology, University of Texas Southwestern Medical Center, Dallas, Texas, USA

**Keywords:** BENIGN INTRACRAN HYP, CSF DYNAMICS, HEADACHE, NEUROOPHTHALMOLOGY

## Abstract

Idiopathic intracranial hypertension (IIH) is a rare but important disease associated with significant morbidity. There is an expected rise in prevalence in line with the escalating global burden of obesity. Modern revisions in the terminology and diagnostic criteria for IIH help guide clinicians in investigations and researchers in standardising recruitment criteria for clinical trials. The pathophysiology of IIH is incompletely characterised; suggested underpinning mechanisms include the role of cerebrospinal fluid regulation as well as metabolic and endocrinological perspectives. Recent treatment trials are providing insights into the management but debate still surrounds key areas in treatment. This review will provide an up-to-date discussion on the potential pathogenic mechanisms and management of IIH.

## Introduction

Idiopathic intracranial hypertension (IIH) is characterised by raised intracranial pressure (ICP) of unknown cause, when all other causes of raised ICP have been excluded.^1^ IIH causes significant morbidity, including permanent visual loss in up to 25% of cases^2^ with reports of 1–2% of new cases being registered blind per year^3^ and disabling headache in the majority.^4^ With peak presentation being between ages 20 and 40 years^5^; an overwhelming female predominance and a strong association with obesity^5^; IIH, is well known as a disorder that affects overweight women of reproductive age.

There are a number of indicators that incidence and prevalence of IIH are rising, likely related to increasing prevalence of obesity worldwide; WHO indicates a twofold increase in the UK between 1997 and 2002 and a threefold increase in the USA between 1990 and 2006. A recent epidemiology study reported an incidence of IIH occurring in 28/100 000/year,^6^ which is the highest reported rate in the literature so far.^7^ In the USA, there was a 320% increase in new cerebrospinal fluid (CSF) shunt procedures for IIH between 1998 and 2002.^8^ IIH is an expensive condition for society and the individual: in the USA, total economic costs are estimated at greater than US$444 million per annum, and 57% of patients report significant lost earnings with 31% changing occupation.^9^

The aetiology and pathology are not fully established, and the disorder has become the subject of increasing scientific scrutiny over the last decade. With no single cause implicated, uncertainties surround the best therapeutics which have led to differing management strategies. Both adults and children can be affected by IIH, however, paediatric IIH may have different pathogenesis and clinical course^10^ and is not discussed in this article. In this review, we review adult onset IIH with focus on the latest developments in both the pathogenic mechanisms and management.

## Epidemiology

The incidence and prevalence of IIH depend on the geographical location of the population studied; Andrews *et al*^7^ highlighted the diversity in the worldwide epidemiology for IIH and cautioned against comparing the rates because of inconsistent definitions of obesity used in various studies. Evidence from the literature suggests an incidence between 1 and 3/100 000/year in the general population. When stratified for reproductive age, female gender and weight, the incidence rises by 12–28/100 000/year.^5–7^ ^11^

## Terminology

The nomenclature and diagnostic criteria used to describe this condition have evolved over time. The first case of IIH, then termed serous meningitis, was probably reported in the 1890s by Henrich Quincke, a German physician, when lumbar puncture (LP) was first introduced. Nonne^11^ ^12^described a series of cases, similar to Dandy,^13^ of raised ICP without tumour termed pseudotumour cerebri. Both series contained cases that we now recognise as raised ICP secondary to a known aetiology. The maturing nomenclature and diagnostic criteria have reflected the advances in investigating patients, particularly neuroimaging and understanding of normal CSF opening pressure measurements, and our increasing appreciation of the clinical course of the disease. Some patients have a mild and self-limiting course, while others experience substantial morbidity from visual loss and persistent headache.

## Pathophysiology

**Figure 1:**
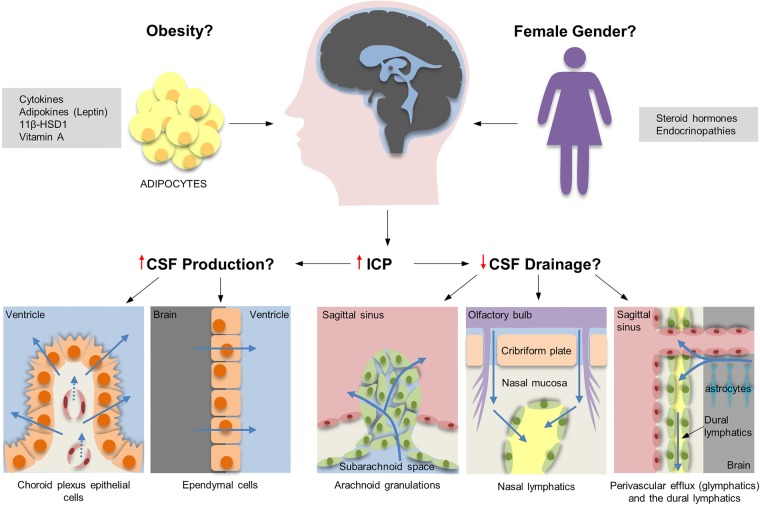
Schematic diagram of the possible pathophysiological mechanisms in idiopathic intracranial hypertension (IIH). Cerebrospinal fluid (CSF) is produced mainly by the choroid plexus epithelial cells, with a small amount being secreted by ependymal cells that line the ventricular system. Classically, CSF was thought to drain predominantly through the subarachnoid space through arachnoid granulations into the superior sagittal sinus. Evidence also suggests CSF drains through the cribriform plate along cranial nerves into the nasal lymphatics (yellow). The most recent hypothesis proposes bulk flow of fluid along perivascular routes (glymphatic pathway) which is cleared from the brain into the subarachnoid CSF, bloodstream or cervical lymphatics. Supporting this concept is the recent discovery of lymphatic vessels (yellow) in the dura that drain into the deep cervical lymph nodes.

The underlying pathogenesis of IIH is uncertain. Raised ICP is a uniform characteristic, but the mechanism by which ICP is elevated in IIH is not clear. It is also questionable whether a single unifying mechanism elevates ICP in these individuals (figure 1). Secondary causative factors which lead to elevation of ICP may be mechanistically distinct from truly idiopathic causes.

### Role of altered CSF dynamics

Changes in the volume of blood, CSF and brain tissue influence ICP. IIH likely represents a disorder of CSF regulation, potentially through CSF hypersecretion or reduced drainage.

### Increased CSF production

The choroid plexus is the primary site of CSF secretion, generating around two-thirds of the total CSF produced, with the rest coming from extrachoroidal sources, such as the ependyma and possibly the blood brain barrier.^14^ ^15^ In the choroid plexus, CSF production is governed by a number of ion transporters located on the choroid plexus epithelial cells. The net movement of ions across these cells results in the movement of water and, thereby, secretion of CSF. Increases in CSF production may be attributed to an increase in choroid plexus size, or increased activity of the choroid plexus. Macroscopically, patients with IIH do not show any hypertrophy of the choroid plexus. Additionally, CSF overproduction has only been demonstrated in cases of choroid plexus papilloma and villous hyperplasia of the choroid plexus, which all produce hydrocephalus. Ventricular size is not altered in IIH, making CSF hypersecretion unlikely. Infusion studies can provide an indicator of CSF secretion. However, there are few studies in this area, and the results are inconsistent.^16^

Dysregulation of fluid transport in IIH may be important. The water transporting channel, aquaporin 4, is localised to the astrocyte foot process, and has a role in fluid transport predominantly relating to cerebral oedema. Anti-aquaporin 4 antibodies have not been identified in IIH,^17^ and there is no evidence of upregulation of the aquaporin 4 gene.^18^ Aquaporin 1 is another candidate as these channels are predominantly in the choroid plexus, can be upregulated by retinoids and glucocorticoids, and are affected by medications used to treat IIH.

### Reduced CSF drainage

Historically, it was believed that CSF drains from the subarachnoid space through arachnoid granulations into the superior sagittal sinus. However, evidence of CSF drainage through the cranial nerves, and the cribriform plate into the lymphatics, has been demonstrated in various species, including humans, using microfil injections.^19^ Recently in mice, lymphatic vessels have been discovered lining the dural sinuses which are then able to drain CSF into the deep cervical lymph nodes.^20^ This latest research disputes the long-held belief that the central nervous system lacks a lymphatic system. In addition, the newly named ‘glymphatic pathway’ suggests that there is an exchange in fluid between the CSF in the subarachnoid space and the interstitial fluid in the brain along paravascular routes (figure 1).^21^ Arachnoid granulation agenesis in children does not always result in elevated CSF, indicating that alternative CSF drainage pathways are functioning. There is very limited understanding of the role of lymphatic and glymphatic drainage in IIH, but these pathways may well be highly relevant.

A reduction in CSF absorption by the arachnoid granulations may be attributed to either an increase in CSF outflow resistance, or a reduction in the pressure gradient between the subarachnoid space and the superior sagittal sinus. Increased CSF outflow resistance has been demonstrated to lead to elevated ICP, as seen in posthaemorrhagic hydrocephalus, where blockage of the arachnoid granulation and lymphatics by blood cells or fibrosis is noted.^22^ Through a similar mechanism, both CSF hypercellularity, as seen in malignant meningitis, and high CSF protein, in Guillian-Barré syndrome, can elevate ICP. Vitamin A deficiency can also lead to elevated ICP with evidence of thickening of the extracellular matrix in the arachnoid villi.^21^ Reduced CSF absorption has also been demonstrated in patients with IIH through isotope methods designed to evaluate CSF circulation and absorption.^23^

### Role of obesity

Obesity is a consistent risk factor for the development of IIH. Correlations between body mass index (BMI) and risk of IIH have been demonstrated,^24^ as have associations between increase in weight and disease recurrence.^25^ Conversely, weight reduction has been observed to improve vision.^26^ This was confirmed in a prospective cohort study using a low-calorie meal replacement to induce weight loss, generating improvements in ICP and papilloedema, as well as symptomatic improvements in headache.^27^

Despite the association between IIH and an obese phenotype, the pathological mechanisms tying the two together are unclear, and IIH is a rare disorder, while obesity is common. Suggestions that centrally distributed adiposity transmits pressure, thereby generating raised ICP, are questionable, as few obese patients have elevated ICP. Additionally, studies of waist:hip ratios in patients with IIH suggest that adiposity is predominantly in the lower body in IIH patients, by contrast with central adiposity of typical obesity.^28^ A number of studies have evaluated cytokine and adipokines profiles in the serum and CSF of patients with IIH, but have been limited by small numbers and suboptimal control groups. Results have not been consistent, which may also be related to differences in the sensitivity of the assays used. Leptin, an adipokine which regulates satiety at the hypothalamus, was elevated in the serum in one study, but this effect was absent when BMI was controlled for.^29^ ^30^ CSF leptin levels appear elevated in IIH compared with controls matched for BMI, age and gender. However, it remains to be established whether dysregulation of adipokines and cytokines are pathogenic in dysregulating ICP, or merely reflect a consequence of the disease.

Retinol (a vitamin A derivative) has also been implicated in IIH. Arctic explorers developed elevated ICP following excessive ingestion of vitamin A rich polar bear liver.^31^ Levels of retinol and retinol-binding protein have been evaluated in the serum and CSF of patients with IIH, with results of both elevated and decreased levels.^32^ ^33^

### Role of gender

A number of case reports have documented patients developing raised ICP in the setting of endocrinopathies and with steroid use (typically corticosteroid withdrawal). Intracranial hypertension in women using hormonal contraception and during pregnancy has been reported but is unsubstantiated, as many of these cases have occurred in women with other risk factors for IIH.^34^ Few studies have systematically evaluated the hormone profiles in IIH. Early studies demonstrating elevated serum oestrone levels were compromised by small numbers and mixed gender cohorts.^35^ A study using radioimmune assays to evaluate cortisol, testosterone, bioavailable testosterone, prolactin, dehydroepiandrosterone sulfate, androstenedione, insulin, aldosterone, oestradiol, follicle stimulating hormone and luteinising hormone, demonstrated that younger patients with IIH had elevated testosterone and androstenedione but a control cohort was not evaluated.^36^

In the ocular ciliary epithelium 11 β-hydroxysteroid dehydrogenase type 1 (11B-HSD1) drives secretion of aqueous humour and 11B-HSD1 inhibitors reduce intraocular pressure.^37^ The ciliary epithelium and the choroid plexus originate from a similar embryological foundation and share secretory mechanisms. In rabbit and human choroid plexus, a functional 11B-HSD1 pathway has been identified, suggesting a potential role in CSF secretion. In patients with IIH, following therapy with a low-calorie diet, ICP falls in correlation with a reduction in 11B-HSD1 activity.^38^

11B-HSD1 is also dysregulated in obesity and the metabolic syndrome and consequently specific inhibitors are being developed as novel therapies.

### Anatomical influence

**Figure 2:**
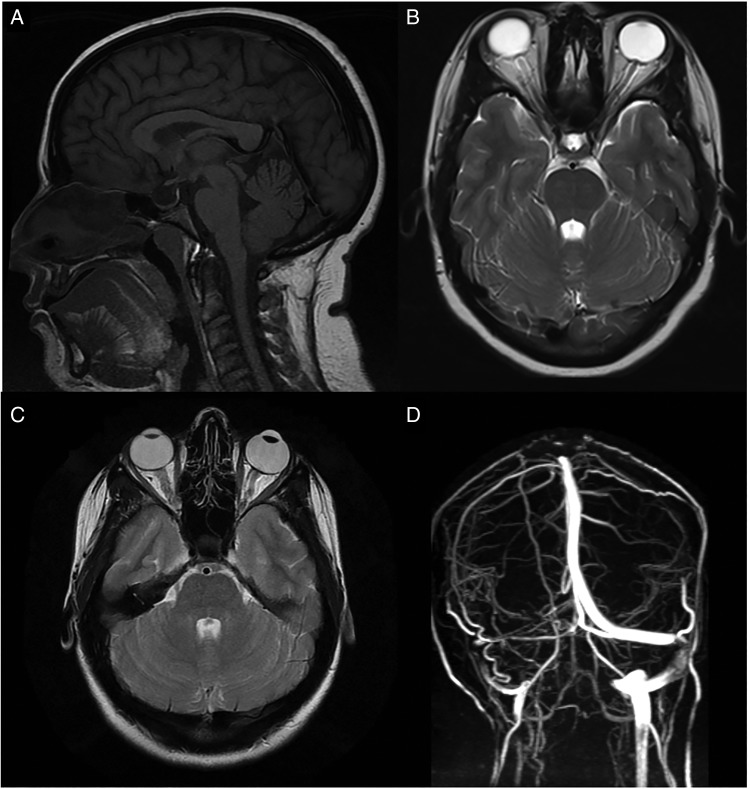
(A) MRI T1-weighted sagittal imaging demonstrating an empty sella (the pituitary gland has been flattened against the wall of the sella). (B) MRI T2-weighted axial image demonstrating flattening of the posterior globes at the insertion of the optic nerves, protrusion of the optic nerve head into the vitreous and increased fluid in the optic nerve sheath complex bilaterally. (C) MRI T2-weighted axial image demonstrating tortuosity (kinking) of the intraorbital optic nerve on the left with fluid in the associated optic nerve sheath complex. (D) MR venography (posterior view) demonstrating a longitudinal extensive left transverse sinus stenosis (extraluminal appearance).

Improvements in brain venography imaging reveal that most patients with IIH have anatomical abnormalities of the cerebral venous sinus system.^39^ These include stenosis of the dominant (figure 2) or both transverse sinuses. There are two recognised morphological types of venous stenosis, and a combination of both can occur in one patient. An extrinsic stenosis is described as a smooth gradually narrowing tapered stenosis. In the intrinsic type, discrete obstructions within the sinus are seen, and these are thought to be due to arachnoid granulations or fibrous septae.^40^ Reducing ICP has led to resolution of stenosis in some patients, suggesting that the stenoses are a result of raised ICP externally deforming the venous sinuses, and not a primarily cause.^41^ Additionally, elevated venous sinus pressure in the setting of venous sinus stenosis, may impair CSF drainage at the arachnoid granulation tissue further exacerbating a cycle of intracranial hypertension. This has led to the rational for transverse sinus stenting. The degree of stenosis does not appear to uniformly correlate with ICP or visual loss.^39^

## Clinical features

Visual loss is the major morbidity in IIH. Historic hospital-based series reported bilateral blindness in up to 10%.^2^ ^42^ More recently, a British Ophthalmological Surveillance Unit study prospectively found 1–2% of newly diagnosed IIH cases become blind annually in the UK.^3^ The majority of patients with papilloedema suffer some form of visual loss, as measured by formal perimetry.^42^ In fulminant cases visual loss is profound, potentially occurring rapidly over a matter of days.^43^

Headache is the most common presenting symptom of IIH, which can be highly heterogeneous, often described as daily, bilateral, frontal or retroocular. Features consistent with migraine, including unilateral throbbing, with nausea and photophobia are also reported.^44^ Back pain, neck pain and radicular pain frequently occur.^45^

Other ophthalmic-related symptoms include transient visual obscurations and diplopia.^10^ ^45^ Transient visual obscurations (TVO) are characterised by transient visual loss or greying out of the vision, and are usually related to postural changes or straining. They tend to last no longer than a minute, and occur in one or both eyes. TVO are thought to be the result of disc oedema causing transient ischaemia at the optic nerve head.

Diplopia occurs in one-third to two-thirds of patients with IIH at presentation.^2^ It tends to be binocular and horizontal, as a consequence of abducens nerve palsy, and can resolve with normalisation of ICP. Monocular diplopia may occur in the presence of severe papilloedema as a result of macular oedema or later due to epiretinal membrane formation.

Pulsatile tinnitus is common and may be unilateral or bilateral.^2^ ^10^ ^42^ Other associated symptoms include mood disturbance and impairments in memory and concentration.

## Diagnostic criteria

The diagnostic criteria continue to move from symptom-based standards to more quantifiable criteria. The most recent proposal by Friedman *et al* (table 1) reflects the more accepted LP opening pressures (OP) of 25 cm CSF or greater in normal weighted adults and 28 cm CSF or greater in children.^1^

**Table 1:** Diagnostic criteria for idiopathic intracranial hypertension (IIH) adapted from Friedman *et al*^1^

Diagnosis of IIH	Diagnosis of IIH without papilloedema
Diagnosis of IIH is definite if the patient fulfils A–E Papilloedema. Normal neurological examination except for sixth cranial nerve abnormalities. Neuroimaging: Normal brain parenchyma without evidence of hydrocephalus, mass or structural lesion, and no abnormal meningeal enhancement on MRI, with and without gadolinium, for typical patients (female and obese), and MRI, with and without gadolinium, and magnetic resonance venography for others; if MRI is unavailable or contraindicated, contrast-enhanced CT may be used. Normal CSF composition. Elevated lumbar puncture opening pressure (≥250 mm CSF in adults) in a properly performed lumbar puncture.	In the absence of papilloedema, a diagnosis of IIH can be made if B–E are satisfied, and in addition the patient has unilateral or bilateral abducens nerve palsy. In the absence of papilloedema or sixth nerve palsy, a diagnosis of IIH can be suggested but not made if B–E are satisfied, and in addition at least 3 of the following are present on neuroimaging: Empty sella. Flattening of the posterior aspect of the globe. Distension of the perioptic subarachnoid space with or without a tortuous optic nerve. Transverse venous sinus stenosis. (See figure 2: MRI findings in IIH)

The diagnosis of IIH is considered probable if A–D are met, but the cerebrospinal fluid pressure is below 250 mm.

They include MRI scan findings consistent with raised ICP (figure 2). Additionally, they have recognised that IIH may occur in the absence of symptoms of elevated ICP and rarely in the absence of papilloedema, so-called IIH without papilloedema (IIHWOP).

There are a number of identifiable causes that give rise to secondary intracranial hypertension such as anaemia, obstruction to venous drainage and exposure to various pharmacological agents, for example, tetracyclines, hypervitaminosis vitamin A and retinoids (table 2). Neuroimaging, either MRI or CT or MRI, must exclude hydrocephalus, structural lesions, abnormal meningeal enhancement and cerebral venous sinus thrombosis.

**Table 2:** Conditions that may cause intracranial hypertension

Pharmacological agents	Systemic conditions
Antibiotics: tetracycline and derivatives, vitamin A derivatives: isotretinoin, all-trans-retinoic acid (for acute promyelocytic leukaemia) Hormonal agents: corticosteroid withdrawal, growth hormone, thyroxine replacement in children Other agents: lithium, nalidixic acid, rofecoib, cimetidine	Haematological: anaemia Respiratory: obstructive sleep apnoea Renal: renal failure Endocrine: obesity, weight gain, polycystic ovarian syndrome, Cushing's disease, Addison's disease, hypoparathyroidism Genetic: turner syndrome Autoimmune: systemic lupus erythematosus Nutritional: hypervitaminosis A Venous: cerebral venous sinus thrombosis, superior vena cava obstruction, increased right-sided heart pressure

### Comorbidities

Polycystic ovarian syndrome (PCOS) is a chronic endocrine condition characterised by menstrual irregularities, ovarian dysfunction, hyperandrogenism and hirsutism. The prevalence of PCOS in women with IIH is reported to be as high as 39–57%,^46^ compared to 7–18% in the general population. Similar to IIH, PCOS is a disorder of women of childbearing age, associated with obesity, high serum leptin levels and low-grade inflammation.

Metabolic syndrome is a collection of risk factors including abdominal obesity, insulin resistance, circulating hypertriglyceridaemia (dyslipidaemia) and hypertension that combined increased the risk of developing type 2 diabetes mellitus and cardiovascular and cerebrovascular disease. The obese phenotype of patients with IIH suggests that these patients may be at risk of metabolic syndrome, however, the evidence is limited. Fasting insulin levels have been found to correlate with BMI in IIH, as would be expected, but have not been compared with obese controls.^36^

## Assessment and investigations

**Figure 3:**
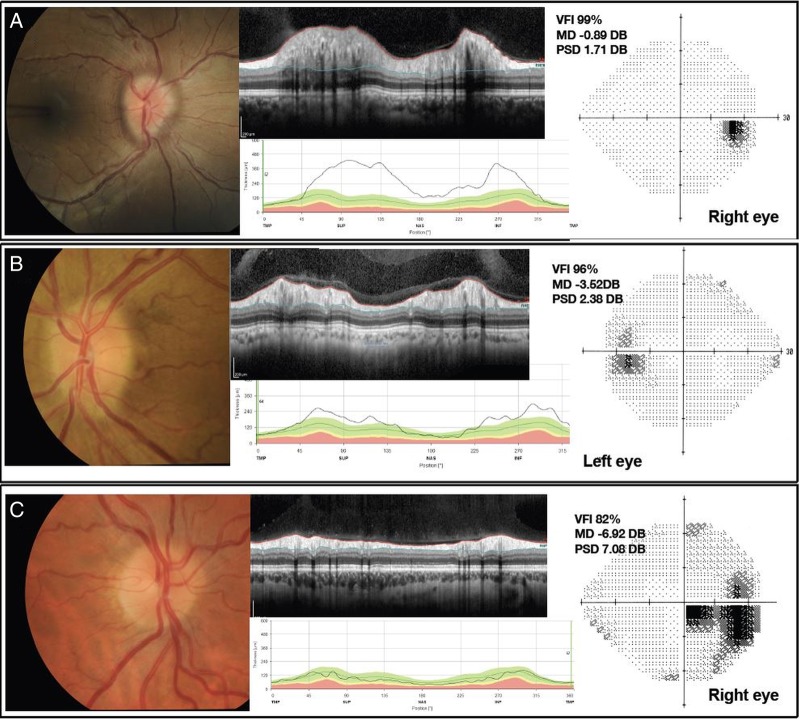
Comprises three patients (A–C). All have a composite of colour fundus photograph on left hand side. Spectral domain (SD) optical coherence tomography (OCT) cross-section image through the optic nerve head, above. The extent on the retinal nerve fibre layer is depicted between the fine red (internal limiting membrane (ILM)) and green lines in this grey scale image. SD-OCT retinal nerve fiber layer (RNFL) thickness line graph showing RNFL thickness values, below. The fine black line depicting the patient's data, and the block colours (green, yellow, red) showing the normative data. Humphrey visual field 24-2 grey scale image on right. VFI, visual field indicator; MD, mean deviation; PSD, pattern SD. Patient A, a newly diagnosed patient with IIH, showing Frisen grade 3, there is obscuration of more than one major vessel leaving the disc, there is a circumferential halo and elevation of all borders. Paton's lines (curvilinear chorioretinal folds adjacent to the temporal (left hand side) of the optic disc margin) are evident. Patient B, a newly diagnosed patient with IIH with Frisen grade 2 there is no major vessel obscured. Circumferential halo and elevation of the nasal border only. Patient C, a treated patient with IIH who has resolved papilloedema (Frisen grade 0). There is reduction in the RNFL thickness which is due to resolution of swelling, but also axonal loss. There is a centrocecal visual field defect.

When papilloedema is identified, it is critical to measure the blood pressure to exclude malignant hypertension. All mandatory tests of visual function should be recorded, including visual acuity, colour vision, pupil examination and a dilated eye examination to document the optic nerve head and macular findings and exclude an ocular cause for bilateral disc swelling. Eye movements should also be tested, as an esotropia at distance may be the only sign of a partial sixth nerve palsy. With papilloedema, the visual acuity may be normal or near-normal, so this measure alone is not predictive of the severity of the disease. Formal visual fields using manual or automated perimetry are required. Papilloedema demonstrates a variety of visual field defects (figure 3) including enlargement of the blind spot, inferonasal defects, as well as central, paracentral, arcuate and altitudinal scotomas.^2^ ^42^ ^42^

Pseudopapilloedema must be distinguished from early papilloedema. Ocular ultrasound may be useful in differentiating optic nerve oedema from drusen and measuring fluid in the optic nerve sheath. Additionally, the calcification in optic nerve head drusen are readily seen on CT imaging, therefore, revisiting imaging performed may be useful. However, we do not recommend drusen as an indication for CT scanning. Fundus fluorescein angiography can identify optic disc leakage in papilloedema, and is used occasionally.^47^

Features of papilloedema include initial hyperaemia of the disc, blurring of the disc margins, elevation of the disc with a periapillary halo, loss of spontaneous venous pulsation and obscuration of major vessels (figure 3). In severe papilloedema, retinal vessels crossing the disc margin and overlying the optic disc are obscured by the oedematous nerve fibre layer; retinal haemorrhages and cotton wool spots may be present, serial colour fundus photographs are useful in recording disc findings and help document resolution.

Formal grading of papilloedema using the Frisén scale has been shown to have a limited reproducibility with high intraobserver and interobserver variability, and requires a senior level of expertise.^48^ Quantification of disc swelling using optical coherence tomography (OCT) is an important development. OCT permits high-resolution non-invasive cross-sectional imaging of the neurosensory retina (figure 3). It is analogous to ultrasound imaging, measuring light waves rather than sound.^49^ Measurement of the following parameters: retinal nerve fibre layer (RNFL) thickness; total retinal thickness; optic nerve (ONH) volume; and retinal ganglion cell layer thickness; for longitudinal monitoring of papilloedema is useful.^50^ Practically in IIH OCT measurements have been shown to correlated with Frisén grade, but not clinical features or visual dysfunction.^50^ However, RNFL thickness demonstrates an indiscriminate ability to separate the resolution of papilloedema from axonal loss in optic atrophy (as exampled in figure 3).^51^ As the technology advances, for example enhanced depth imaging and newer light sources, OCT will hopefully be able to distinguish buried ONH drusen.

Brain imaging has a central role in excluding space-occupying lesions, obstructive hydrocephalus and cerebral venous sinus thrombosis once papilloedema has been diagnosed. Ideally, MRI head and orbits with intravenous contrast and MR or CT venography should be performed. Figure 2 shows commonly observed radiological signs of raised ICP although none are pathognomonic of IIH.

Following brain imaging, LP is mandatory to record the CSF opening pressure and exclude a secondary cause. It is performed with the patient relaxed in the lateral decubitus position. The procedure may be technically challenging in the obese population, and occasionally fluoroscopic guidance is required. CSF from the diagnostic LP is sent for microscopy, measurement of protein, glucose and, if indicated, cytology, culture and assessment of xanthochromia. Serum should be sent simultaneously for glucose, renal function and blood count to exclude anaemia. Further practical advice on investigating IIH can be found in Mollan *et al*.^47^

### Management

The 2015 Cochrane review concluded that there is no current consensus on the best management strategy for IIH.^12^ The two key approaches in IIH are to preserve visual function, and to reduce long-term headache disability. The treatments employed depend largely on the patient's level of visual function and rate of progression. Accepted medical interventions range from dietary therapy (eg, responsible and sustainable weight loss, lifestyle modification, low-salt diet) to medications and surgical treatment.

### Weight loss

Newborg^51^ was the first to document diet as a treatment for IIH. In her uncontrolled prospective case series, nine patients were treated with a low-calorie rice diet with fluid (750–1250 mL/day) and sodium (<100 mg/day) restriction; the resultant weight loss (13–38%) was associated with improvement in IIH symptoms and assessments of papilloedema. The association between weight loss and improvement in papilloedema was further supported by several retrospective case reviews.^52^ ^53^

A low-salt diet is often recommended in IIH based on the association with orthostatic oedema in women.^54^ However, there is no direct evidence for the mechanism of action of salt restriction in vivo to reduce ICP.

On the basis of small studies showed that reduction of body weight by 6% was associated with improved visual function and papilledema grade in IIH,^32^ ^52^ weight loss was studied in a prospectively designed cohort study.^27^ Patients with chronic IIH were treated with a severely restricted calorie-controlled diet (425 kcal/day) for 3 months following a 3-month observational period; 44% of the patients took a stable dose of acetazolamide during the study. Internal comparators prediet and postdiet within the same patient demonstrated significantly reduced ICP measurements (−8 cm CSF, p<0.001), headache scores and papilloedema. There was a 15% mean weight reduction (p<0.001). All qualitative and quantitative improvements were sustained at 3 months postdiet cessation. This study provided evidence that weight loss can lower ICP.

Long-term maintenance of weight loss is notoriously difficult and is typically as little as 2–4 kg at 2 years, irrespective of the dietary regime followed.^55^ In keeping with this, patients in the Birmingham weight loss study^27^ were noted to regain weight in the year following discontinuation in the study and, consequently, their symptoms and signs of IIH relapsed, a documented phenomenon in IIH.^25^ ^42^

Obesity pharmacological therapies, such as tetrahydrolipstatin and phentermine/topiramate may induce a modest weight loss, but have not been specifically studied in IIH.^55^ There is class IV evidence for bariatric surgery as an effective treatment for IIH in obese patients, both in terms of symptom resolution and visual outcome.^55^ A randomised control trial evaluating bariatric surgery in IIH is currently underway (Clinical trials. gov ID NCT02124486).

### Pharmacotherapy

#### Acetazolamide

Acetazolamide is currently considered the mainstay of pharmacological management in IIH. It is a potent enzyme inhibitor of carbonic anhydrase, and it impedes the activity at the choroid plexus reducing CSF secretion. A recent Cochrane systematic review identified two randomised control trials for the use of acetazolamide in IIH.^12^

Ball *et al*^57^ in the UK, prospectively evaluated acetazolamide use in mild IIH. Fifty patients were recruited and randomised to receive acetazolamide (daily dose between 250 and 1500 mg) or placebo. Symptoms, body weight, visual function and quality-of-life measures were recorded. Regardless of treatment allocation, all overweight patients were advised to follow a weight reduction programme. There was no significant difference between the treatment arms at the final 12-month follow-up evaluation; based on this study design, a sample size of 320 would be required to demonstrate a 20% treatment effect. Overall, most participants showed clinical improvement, with 44% judged to be in remission at trial end. Mean weight loss was 6.2 (5.8%) and 3.3 kg (3.5%) in the treatment arm and control arm, respectively. They highlighted issues in conducting a trial in IIH that included recruitment, choice of outcome measures and adherence to acetazolamide.

The Neuro-Ophthalmologic Research Disease Investigator Consortium (NORDIC) enrolled 165 participants to the IIH Treatment Trial (IIHTT).^58^ This was a multicentre, randomised, double-masked, placebo-controlled trial designed to determine the efficacy of acetazolamide compared with placebo in IIH, with both arms also receiving a supervised weight-reduction, low sodium diet. The initial dosing of acetazolamide was 500 mg twice a day with a schedule to increase the dose by 250 mg every 6 days up to a maximum of 4 g daily. Participants who could not tolerate the study drug could decrease the dosage to a minimum of 125 mg/day. All participants received a specific dietary plan and lifestyle modification programme. The primary outcome measure was the change in perimetric mean deviation (PMD) on automated visual field analysis at 6 months. The study population included individuals with IIH and mild visual field loss, defined as PMD of −2 to −7 dB in the worst eye^59^ (figure 3 shows two patients (B and C) visual fields that fall between −2 and −7 dB).

Both treatment groups in the IIHTT showed improvement in the primary outcome measure of PMD from baseline (−3.53 dB in both groups). At 6 months, the PMD in the acetazolamide group had improved more than the placebo group (−2.10 dB compared to −2.82 dB, a difference of 0.71 dB, p=0.05). Significant difference in improvements between the acetazolamide and placebo group was also reported for the fellow eye PMD, Frisén papilloedema grade, CSF opening pressure and quality-of-life measures including the Visual Function Questionnaire-25 and its neuro-ophthalmic supplement. No significant difference between the groups was found in visual acuity in the study or fellow eye, headache or headache disability.

Both groups lost weight at 6 months from a baseline of 107.72 kg. Those receiving acetazolamide lost more weight (−7.50 kg) than those receiving placebo (−3.45 kg), and this difference was highly significant (p<0.001). The mean dose of study drug in the acetazolamide group at conclusion of the study was 2.5 g/day, which is in excess to what is normally prescribed in routine UK clinical practice due to side effects. Indeed, in the IIHTT, adverse effects of fatigue, nausea, diarrhoea, vomiting and paraesthesia were significantly higher in the acetazolamide group. There were 6 treatment failures in the placebo group and only 1 in the acetazolamide group. 19% of subjects withdrew from the study but the reasons and frequency of withdrawal was similar in both groups. The IIHTT provides class 1 evidence that acetazolamide use provides a modest improvement in visual field function in patients with IIH with mild loss.

### Topiramate

Topiramate alters several targets such as inhibition of voltage-gated sodium and calcium channels, augmentation of γ-aminobutyric acid (GABA)-induced chloride flux, inhibition of glutamate-related excitatory neurotransmission and action on carbonic anhydrase isoenzymes. Topiramate use was first reported in a prospective open label study of 40 patients using both topiramate (daily dose range 100–150 mg) and acetazolamide daily (dose range 1000–1500 mg)^60^; however, no placebo group was included. A statistically significant improvement in visual field was detected with both drugs, with no statistically significant difference found when compared with each other. Weight loss was prominent in the topiramate group. The side effect profile is similar to acetazolamide, however, adverse cognitive effects can occur, and the drug is not recommended for use in patients with a history of severe depression. Additionally, as topiramate is a first-line agent for migraine prevention (National Institute for Clinical Excellence Guideline CG150) it may have additional benefit for those patients with IIH with coexisting migraine-like headaches (68% at diagnosis).^44^

### Other drugs

Other diuretics, such as furosemide, are used in IIH, when acetazolamide is contraindicated or not tolerated. There is animal evidence that furosemide reduces CSF production, possibly by a different mechanism to acetazolamide, and it is postulated that it might have an additive effect to acetazolamide.^61^ Caution is advised when combining diurectics, as severe hypokalaemia may result. Octreotide is a somatostatin analogue which inhibits pituitary growth hormone (GH) secretion, and antagonises GH and insulin-like growth factor action by blocking GH receptors. Somatostatin receptors are highly expressed in arachnoid villi and choroid plexus and, therefore, may be related to CSF production and absorption. Following case reports, an unblinded, open-label study reported resolution of papilloedema in 92% of cases with further improvement in other visual parameters and headache.^62^ Given the observational nature of this study, the small study size, and the lack of a control group, further data are required.

### Non-medical interventions

If medical therapy fails, is not tolerated, or there is fulminant IIH, more aggressive measures should be considered. Management options include serial LP; optic nerve sheath fenestration (ONSF); neurosurgery employing shunts and stenting of the transverse venous sinuses. The decision regarding the choice of surgical procedure often depends on local expertise and the patient's morbidity.

### Lumbar puncture

Occasionally, a single LP may be all that is required to put IIH into remission.^63^ Serial LP are useful temporising measures while awaiting surgery, or in pregnant patients who wish to avoid medical therapy. LP may reverse transverse venous sinus collapse by lowering CSF pressure, therefore, providing temporary relief until the sinus re-collapses, usually within weeks. Complications include low-pressure headaches, CSF leak and CSF infection.

### Optic nerve sheath fenestration

ONSF aims to relieve raised CSF pressure in the subarachnoid space surrounding the optic nerves by incision of the meninges enclosing the optic nerve. It is regarded by some as the preferred option for patients with visual symptoms, particularly if there is unilateral visual compromise. A recent meta analysis included 712 patients who underwent ONSF: 59% had an improvement in vision, 44% resolution of headache, and 80% reduction in papilloedema.^64^ Complications included diplopia and pupil abnormalities with permanent visual loss reported in 1–2% secondary to retinal vascular occlusion or traumatic optic neuropathy. Unilateral ONSF has been reported to significantly improve papilloedema grade in both eyes. Repeat surgery was required in 14.8% of patients, and over a third of patients required subsequent CSF diversion.^64^

A comparative case series between ONSF and CSF diversion showed both procedures resulted in improved visual acuity and visual fields (using PMD) postsurgery, but CSF diversion resulted in a statistically significant improvement in PMD compared to ONSF. Lack of randomisation and selection bias limit the utility of this study.^65^

### CSF diversion surgery

CSF diversion surgery was initially employed in 1949 using ventriculoperitoneal shunts, and by the 1970s, good outcomes were reported with lumboperitoneal shunting (LPS). In LPS, a catheter inserted into the subarachnoid space at the level of the lumbar spine is then tunnelled under the skin, continuing around the oblique muscles and into the peritoneal cavity where the drained CSF is absorbed. Shunts with a valve system and CSF reservoir are recommended as they reduce risk of significant pressure fluctuations. Complications of CSF diversion surgery include shunt infection, shunt obstruction, intra-abdominal pain and CSF leak. Rarer complications include cerebellar tonsillar herniation and syringomyelia, subdural and subarachnoid haemorrhage, and bowel perforation. Shunt revisions have been reported in 51% of patients and multiple revisions required in 30%.^66^ Sinclair *et al*^66^ found significant improvements in visual acuity maintained postoperatively at 6 (p=0.002) and 12 months (p=0.016), but headache symptoms persisting in the majority of patients at 12 months.

### Transverse sinus stenting

Teleb *et al*^67^ reported the largest group analysis to date for the endovascular management of IIH. The gradient threshold at which the transverse sinus stentings (TSS) were performed varied between ≥4 to ≥10 mm Hg. Antithrombotic therapy was typically dual antiplatelet therapy (aspirin and clopidogrel). Patients were pretreated and expected to remain on dual therapy for up to 6 months, followed by aspirin for up to 12 months. Complications of the procedure include a short-lived ipsilateral headache in many, restenosis and, in rare cases, vessel perforation leading to acute subdural haematoma, stent migration and thrombosis. TSS conferred functional improvements in symptoms and signs including headache, papilloedema grade and reported visual loss. There are currently major limitations of evidence with case series being non-randomised, not detailing morphological stenosis type, being small in size with selection bias and a lack of long-term follow-up.^68^ At least one death has been reported, with known cases of life-threatening haemorrhage and herniation following stenting.

### Treatment of headache

Headache is the most common presenting feature in IIH and leads to significant morbidity.^69^ Many patients do not have the classical headache phenotype attributed to raised ICP and the International Classification of Headache Disorders, which does not specify a particular headache phenotype (ICHD 3) has poor specificity (53%) in IIH.^70^ Many patients with IIH have daily headache (86%) that often resembles migraine or tension-type headache. Other features from the history can be useful, but are not pathognomonic, and can occur in both IIH and headache control subjects (exacerbation with bending 50% and 44%, respectively; morning headache 20% and 29%, respectively; exacerbation with cough or Valsalva 70% and 35%, respectively).^70^ IIH headache improves post-LP in 72%, but an improvement also occurs in 25% of those with headache without IIH or a pressure syndrome.^70^

Most headache improvement occurs during the first month following diagnosis and treatment with constant headaches in 64% at diagnosis improving to 13% by 1 month.^44^ Yet, 63% continue to have headaches at 12 months, and headaches can remain even after ICP has normalised in 67%. Headaches outcomes are poor even in those with no prior history of headache, with 57% having headaches at 12 months.^44^

There is little evidence to guide the management of headache attributed to IIH. Accurate headache phenotyping is key, and mixed headache types frequently coexist (high pressure, migraine, tension-type, low pressure in those with a shunt). Medication overuse headaches often occur and are underdiagnosed.^71^

Our practice is to treat the predominant headache phenotype, typically using migraine-preventative strategies. It is worthy of note that some of the commonest medications here can cause weight gain. An excellent review in this area covers these options in detail.^72^ Shunting is generally ineffective management strategy for raised ICP headaches, as 79% still have headaches at 2 years, low-pressure headaches may develop (28%), and shunt failure is a major issue (>50% are revised).^66^ Weight loss strategies significantly improve headache.^30^ In the IIHTT, headache disability improved in both treatment groups with no benefit of acetazolamide compared with placebo.^58^ As headaches have such a significant impact on patients, management by a headache medicine specialist is recommended.

### Health-related quality of life

For patients with IIH, the journey is typically extremely challenging. The diagnostic process with urgent brain imaging and LP is frightening and painful. The ensuing discussion about the causative role for obesity may be insensitively handled by the busy physician and can be psychologically damaging for the patients. For many, the reality is a long-term illness dominated by drug side effects, headaches, potential depression and in some surgery, which can be recurrent. The impact on their family life, occupation and earning potential is great.

Studies verify that IIH has a significant effect on health-related quality of life (QOL)^69^ ^73^ patients with IIH have also been found to have higher levels of anxiety, depression and fatigue than controls.^74^ In the IIHTT QOL scores were measured and found to be reduced at the time of the IIH diagnosis.^75^ PMD in the best eye, visual acuity in the worst eye, visual symptoms, and pain symptoms, but not obesity, were independently associated with reduced QOL. QOL scores have been shown to improve with weight loss alongside significant improvement in clinical measures and headache, in IIH. In this study, headache disability was the only clinical outcome that correlated with impaired QOL, a finding also noted in other studies.^73^

### Cognition

A number of factors could impact on cognitive function in IIH (depression, headaches, sleep apnoea, obesity, medications and raised ICP). One study reported reduced reaction time and processing speed in patients with acute IIH compared with age and gender-matched controls. These deficits were maintained at a 3-month follow-up, despite improving headaches and ICP.^75^ Another cross-sectional study has also demonstrated multidomain mild cognitive impairment in IIH, with lowest scores in visuospatial, attention and global indices.^76^ Cognitive dysfunction may contribute to the patient morbidity, however, it is largely uncertain whether the deficits are chronic or related to treatment effects.

### IIH without papilloedema

In patients with chronic daily headache, raised CSF pressure has been identified in some patients, particularly in the obese, despite the lack of papilloedema. The chosen cut-off for ‘normal’ ICP is essential when considering the diagnosis of IIHWOP. Earlier series using an LP pressure cut-off value of 20 cm CSF are likely to have led to overestimation of the number of cases.^77^ Although, patients with IIWOP have been found to have lower LP opening pressure than those with papilloedema (31 vs 37 cm CSF), are more likely to be treatment-resistant, and have non-organic visual field defects.^78^ The latest diagnostic criteria (table 1) have helped to establish which patients may truly have raised ICP by specifying the need for identification of ancillary features of raised ICP (imaging features and sixth cranial nerve palsies). Pulsatile tinnitus is a feature of raised ICP and is more common in patients with IIH than migraineurs, but can occur in both patient cohorts (64% compared against 26%).^70^

Isolated, single LP measures are not ideal in assessing patients with chronic headaches as ICP fluctuates diurnally. In some cases, a period of prolonged CSF monitoring, ideally while the patient is ambulatory is helpful.^79^ Accurate diagnosis is essential to ensure chronic migraine with a borderline-raised ICP are not incorrectly labelled as having IIHWOP.

The absence of papilloedema, despite elevated ICP is intriguing and requires further evaluation. Anatomical variations in the optic canal diameter are seen even within the same patient leading to asymmetrical papilloedema which has been elegantly illustrated by Bidot.^80^

As patients with IIHWOP do not have papilloedema and do not develop papilloedema, they do not develop visual loss (although functional visual field constriction occurs in 20%).^77^ Consequently, visual monitoring is not necessary and management should focus on the headache. Venous sinus stenoses have been identified in these patients as would be consistent with a raised pressure syndrome. However, the role of venous stenting in this cohort is not established, particularly as venous sinus stenosis can occur in healthy asymptomatic individuals, in migraineurs and in asymptomatic individuals labelled with IIHWOP.^81^

## Conclusion

IIH is classified as a rare condition, and early diagnosis and treatment are imperative to prevent permanent visual loss. The burden on the health service is significant and costly due to high healthcare utilisation by patients. This is unlikely to improve in the setting of growing global obesity figures. For clinicians managing patients with IIH, questions remain about the optimal evidence-based approach for management. The conundrum of how obesity and female gender contribute to the underlying aetiology remains. IIH is a multisystem disorder encompassing neuroscience, ophthalmology and endocrinology and cross-specialty collaboration will be integral to advance our understanding of IIH. In the clinical environment, multidisciplinary input is essential to optimise patient care.

Recent research momentum has led to important progress in our understanding and management of IIH. The field continues to advance with a number of ongoing trials (eg, IIH:WT evaluating weight loss strategies and bariatric surgery (NCT02124486), and IIH:DT evaluating a novel 11β HSD1 inhibitor (NCT02017444), stenting in refractory IIH (NCT02143258)), and studies the NORDIC group evaluation of vitamin A and genetics). The results of these studies are eagerly anticipated, and will, hopefully, translate into improved patient care.
